# Equilibrium Thermodynamic Properties of Aqueous Solutions of Ionic Liquid 1-Ethyl-3-Methylimidazolium Methanesulfonate [EMIM][MeSO_3_]

**DOI:** 10.1038/s41598-020-59702-z

**Published:** 2020-02-21

**Authors:** Chaolun Zheng, Jian Zhou, Yong Pei, Bao Yang

**Affiliations:** University of Maryland, Department of Mechanical Engineering, College Park, MD 20742 USA

**Keywords:** Mechanical engineering, Materials science

## Abstract

The ionic liquid 1-ethyl-3-methylimidazolium methanesulfonate ([EMIM][MeSO_3_]) has been considered as a promising alternative desiccant to triethylene glycol and lithium bromide commonly used in the industry. In this paper, the water activity coefficient of this binary system was measured from 303 K to 363 K with water concentration from 18% to 92%. The interaction energies between the ionic liquid molecules (${g}_{22}$) and between the ionic liquid and water molecules (${g}_{12}$) for the [EMIM][MeSO_3_]/water binary system were determined from the water activity coefficient data using the Non-Random Two-Liquid (NRTL) model. The magnitude of the interaction energy between the [EMIM][MeSO_3_] and water molecules (${g}_{12}$) was found to be in the range of 45~49 kJ/mol, which was about 20% larger than that between the water molecules (${g}_{11}$) in the [EMIM][MeSO_3_]/water system. The large (${g}_{12}$) can explain many observed macroscopic thermodynamic properties such as strong hygroscopicity in the ionic liquid [EMIM][MeSO_3_]. These interaction energies were used to determine the heat of desorption of the [EMIM][MeSO_3_]/water system, and the obtained heat of desorption was in good agreement with that calculated from the conventional Clausius-Clapeyron Equation.

## Introduction

Ionic liquids are compounds composed of organic cations and inorganic anions, and they show a negligible vapor pressure and good fluidity over a wide temperature range^1,2^. Some ionic liquids have been found to be highly hygroscopic, which makes them promising desiccants for applications in gas dehydration and absorption cooling. In contrast to triethylene glycol currently used in gas dehydration, losses by evaporation can be eliminated when the ionic liquids are used as desiccants. In addition, the use of ionic liquids can avoid the crystallization and corrosion problems^3,4^, which are the two major concerns of the most commonly used halide salt liquid desiccants^5,6^.

The ionic liquid 1-ethyl-3-methylimidazolium methanesulfonate ([EMIM][MeSO_3_]) is among those that are highly hygroscopic, and is a promising candidate for next generation of desiccants^7,8^. The determination of thermodynamic properties is important for performance evaluation for this special ionic liquid. Many researchers have conducted experiments to measure the important macroscopic thermodynamic properties, such as specific heat^9,10^, density^7,9–12^, viscosity^7,9,11,13^, electrical conductivity^9^, surface tension^11,14^, reflective index^11^, diffusion coefficient^7^, nuclear magnetic resonance (NMR) spectroscopy^9,14,15^, excess molar heats of mixing^9,12,16^, and water activity coefficient^7,16^.

Although the macroscopic thermodynamic properties of the [EMIM][MeSO_3_]/water binary system have been extensively investigated, the molecular thermodynamic properties of this binary system are not comprehensive. For example, the interaction energy or bonding energy between the ionic liquid molecules, and between the ionic liquid and water molecules are extremely scarce for the ionic liquid [EMIM][MeSO_3_]/water systems. These molecular interaction energy properties are related to many macroscopic thermodynamic properties such as heat of desorption, heat capacity, hygroscopicity, and water vapor pressure.

In this work, the Non-Random Two-Liquid (NRTL) model was used to determine the interaction energies between different molecule pairs inside the [EMIM][MeSO_3_]/water binary system from the measured water activity coefficients data. These interaction energies were used to determine the heat of desorption of the [EMIM][MeSO_3_]/water system, which was in good agreement with those calculated from the Clausius-Clapeyron Equation. A formula to predict heat of desorption from the interaction energies was also developed for the binary systems.

## Theoretical Background

### Water activity coefficient

The activity coefficient of water ${\gamma }_{{{\rm{H}}}_{2}{\rm{o}}}$ is a fundamental thermodynamic parameter that accounts for deviations from ideal behavior in non-ideal solutions, such as the aqueous ionic liquid solutions, which is defined as^7,8^:

1 $${\gamma }_{{{\rm{H}}}_{2}{\rm{O}}}=\frac{{p}_{{{\rm{H}}}_{2}{\rm{O}},{\rm{non}} \mbox{-} {\rm{ideal}}}}{{p}_{{{\rm{H}}}_{2}{\rm{O}},{\rm{ideal}}}}=\frac{{p}_{{{\rm{H}}}_{2}{\rm{O}},{\rm{non}} \mbox{-} {\rm{ideal}}}}{{x}_{{{\rm{H}}}_{2}{\rm{O}}}\cdot {p}_{{{\rm{H}}}_{2}{\rm{O}},{\rm{sat}}}}$$

where ${p}_{{{\rm{H}}}_{2}{\rm{O}},{\rm{ideal}}}$ and ${p}_{{{\rm{H}}}_{2}{\rm{O}},{\rm{non}} \mbox{-} {\rm{ideal}}}$ are the partial pressure of water above the ideal and non-ideal aqueous solutions, respectively, $\,{p}_{{{\rm{H}}}_{2}{\rm{O}},{\rm{sat}}}$ is the saturation pressure of pure water, and ${x}_{{{\rm{H}}}_{2}{\rm{O}}}$ is the molar fraction of water in the aqueous solutions. In Eq. (1), the ratio ${p}_{{{\rm{H}}}_{2}{\rm{O}},{\rm{non}} \mbox{-} {\rm{ideal}}}/{p}_{{{\rm{H}}}_{2}{\rm{O}},{\rm{sat}}}$ is the relative humidity (RH) of the non-ideal aqueous solutions. In the ideal aqueous solutions, the partial pressure of water can be described by Raoult’s law^7^:

2 $${p}_{{{\rm{H}}}_{2}{\rm{O}},{\rm{ideal}}}={x}_{{{\rm{H}}}_{2}{\rm{O}}}{p}_{{{\rm{H}}}_{2}{\rm{O}},{\rm{sat}}}$$

However, the [EMIM][MeSO_3_]/water solution is a non-ideal solution, in which the interaction energy between [EMIM][MeSO_3_] and water is significantly stronger than those between water and water. A small value of water activity coefficient ${\gamma }_{{{\rm{H}}}_{2}{\rm{O}}}$ indicates a large intermolecular force between water and ionic liquid molecules and strong hygroscopicity for water absorption. The water activity coefficient at infinite dilution ${\gamma }_{{{\rm{H}}}_{2}{\rm{O}},\infty }$ is often used for the comparison of the hygroscopicity or absorption strength of different desiccants^7^.

### Non-random two-liquid (NRTL) model

The NRTL model^17–20^ correlates the activity coefficients ${\gamma }_{i}$ of a compound *i* with its mole fractions ${x}_{i}$ in the liquid solutions:

3 $$\mathrm{ln}\,{\gamma }_{1}={x}_{2}^{2}(\frac{\Delta {g}_{12-11}}{RT}\frac{\exp (\,-\,2{\alpha }_{12}\Delta {g}_{12-11}/RT)}{{[{x}_{1}+{x}_{2}\exp (-{\alpha }_{12}\Delta {g}_{12-11}/RT)]}^{2}}+\frac{\Delta {g}_{12-22}}{RT}\frac{\exp (\,-\,{\alpha }_{12}\Delta {g}_{12-22}/RT)}{{[{x}_{2}+{x}_{1}\exp (-{\alpha }_{12}\Delta {g}_{12-22}/RT)]}^{2}})$$

4 $$\mathrm{ln}\,{\gamma }_{2}={x}_{1}^{2}(\frac{\Delta {g}_{12-22}}{RT}\frac{\exp (\,-\,2{\alpha }_{12}\Delta {g}_{12-22}/RT)}{{[{x}_{2}+{x}_{1}\exp (-{\alpha }_{12}\Delta {g}_{12-22}/RT)]}^{2}}+\frac{\Delta {g}_{12-11}}{RT}\frac{\exp (\,-\,{\alpha }_{12}\Delta {g}_{12-11}/RT)}{{[{x}_{1}+{x}_{2}\exp (-{\alpha }_{12}\Delta {g}_{12-11}/RT)]}^{2}})$$

where the subscripts 1 and 2 refer to component 1 and component 2 in the binary solution, respectively, $\Delta {g}_{12-11}$ and $\Delta {g}_{12-22}\,$are the exchange in the interaction energy between molecules, ${\alpha }_{12}$ is the non-randomness parameter, *R* is the molar gas constant, and *T* is the absolute temperature. In the case of infinite dilution, the NRTL equations reduce to

5 $$\mathrm{ln}\,{\gamma }_{1,\infty }=\frac{\Delta {g}_{12-11}}{RT}+\frac{\Delta {g}_{12-22}}{RT}\exp (\,-\,{\alpha }_{12}\Delta {g}_{12-22}/RT)$$

6 $$\mathrm{ln}\,{\gamma }_{2,\infty }=\frac{\Delta {g}_{12-22}}{RT}+\frac{\Delta {g}_{12-11}}{RT}\exp (\,-\,{\alpha }_{12}\Delta {g}_{12-11}/RT)$$

The water activity coefficient at infinite dilution ${\gamma }_{{{\rm{H}}}_{2}{\rm{O}},\infty }$ can be used to compare the hygroscopicity or absorption strength of different desiccants.

In the NRTL model, the exchange in the interaction energy $\Delta {g}_{12-11}={g}_{12}-{g}_{11}$, which is the interaction energy change as a result of breaking a 1-1 interaction ${g}_{11}$ and forming a 1–2 interaction ${g}_{12}$. In this study, components 1 and 2 refer to the water and the [EMIM][MeSO_3_], respectively. So ${g}_{11}$, $\,{g}_{22}$, and ${g}_{12}$ are the interaction energies between the water molecules, between the ionic liquid [EMIM][MeSO_3_] molecules, and between the water and [EMIM][MeSO_3_] molecules. The interaction energy parameters $\Delta {g}_{12-11}$ and $\Delta {g}_{12-22}$ can be determined by data fitting using Eqs. (3) and (4) if the water activity coefficients ${\gamma }_{{{\rm{H}}}_{2}{\rm{O}}}$ with its mole fraction ${x}_{{{\rm{H}}}_{2}{\rm{O}}}$ can be measured in the [EMIM][MeSO_3_]/water binary system. The interaction energy between the ionic liquid and water molecules ${g}_{12}$ can be determined using the formula: ${g}_{12}=\Delta {g}_{12-11}+{g}_{11}$ if the interaction energy between water molecules ${g}_{11}$ is known. Similarly, the interaction energy between the ionic liquid molecules ${g}_{22}$ can be determined by ${g}_{22}=\Delta {g}_{12-22}+{g}_{12}$. These molecular interaction energy properties are related to many macroscopic thermodynamic properties such as heat of desorption, heat capacity, hygroscopicity, and water vapor pressure. One application of these molecular interaction energies is that they can be used to predict the heat of desorption of the aqueous ionic liquid solutions with water concentration from 0% to 100%. In comparison, the Clausius-Clapeyron Equation determines the heat of desorption at the water concentration where the vapor pressure and temperature are known.

### Clausius-Clapeyron equation

The Clausius-Clapeyron Equation relates the vapor-liquid equilibrium (VLE) data (*p* and *T*) to the thermodynamic property, enthalpy of vaporization ($\Delta {H}_{v}$), which is given by^21^

7 $$\mathrm{ln}(p)=-\,\frac{\Delta {H}_{v}}{RT}+{\rm{C}},$$

where $p$ is the vapor pressure at the temperature *T*, $\Delta {H}_{v}$ is enthalpy of vaporization, *R* is the molar gas constant, and ${\rm{C}}$ is a constant. In Eq. (7), $\Delta {H}_{v}$ is assumed to be independent of *T*. However, the temperature dependence of $\Delta {H}_{v}$ cannot be overlooked in the water and [EMIM][MeSO_3_] binary solutions due to the complex interaction between water and [EMIM][MeSO_3_]^21,22^. In a moderate temperature range, $\Delta {H}_{v}$ can be assumed to change linearly with *T*,

8 $$\Delta {H}_{v}=\Delta {H}_{v,0}+aT,$$

where $a$ is the temperature coefficient. So the modified Clausius–Clapeyron Equation for the ionic liquid solutions can be written as follows:

9 $${\rm{l}}{\rm{n}}(\frac{p}{{p}_{0}})=-\,\frac{\Delta {H}_{v,0}}{R}(\frac{1}{T}-\frac{1}{{T}_{0}})+\frac{a}{R}\,{\rm{l}}{\rm{n}}(\frac{T}{{T}_{0}}).$$

Therefore $\Delta {H}_{v}$ can be determined from Eqs. (8) and (9) when the VLE data are measured.

### Uncertainty calculation

The experimental uncertainty in this experiment is estimated using the root-sum-square method suggested by Moffat^23^:

10 $${\rm{\delta }}R={\{\mathop{\sum }\limits_{{\rm{i}}=1}^{{\rm{N}}}{(\frac{\partial R}{\partial {x}_{i}}{\rm{\delta }}{x}_{{\rm{i}}})}^{2}\}}^{1/2}$$

In particular, whenever the equation describing the result is a pure “product form”, as shown in Eq. (11):

11 $$R={X}_{1}^{a}{X}_{2}^{b}{X}_{3}^{c}\,\cdots \,{X}_{M}^{m}$$

the relative uncertainty can be calculated by Eq. (12):

12 $$\frac{\delta R}{R}={\{{(a\frac{\delta {X}_{1}}{{X}_{1}})}^{2}+{(b\frac{\delta {X}_{2}}{{X}_{2}})}^{2}+\cdots +{(m\frac{\delta {X}_{M}}{{X}_{M}})}^{2}\}}^{1/2}$$

## Experimental Methods

### Materials

The 1-ethyl-3-methylimidazolium methanesulfonate ([EMIM][MeSO_3_]) was purchased from Sigma-Aldrich (purity higher than 95 wt. %). Deionized water was used in the experiment.

### Experiment setup

**Figure 1:**
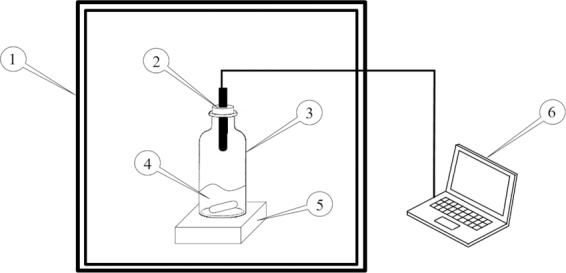
Schematic of the experimental setup for the VLE Measurements. Legend: 1. temperature-controlled oven; 2. humidity sensor; 3. reagent bottle; 4. [EMIM][MeSO_3_]/water solution; 5. magnetic stirrer; 6. DAQ/computer.

The experiment setup for the vapor-liquid equilibrium (VLE) measurement is shown in Fig. 1. The temperature during experiments was controlled using a temperature-controlled oven (Yamato, DKN-402C). The [EMIM][MeSO_3_]/water solution was placed inside a reactant bottle, in which a humidity sensor (Rotronic HC2A-SM) was attached. The RH and temperature (*T*) of the gas phase inside the reactant bottle were measured simultaneously using this humidity sensor with an uncertainty of *T* = ±0.1 K and RH = ±0.8% of the RH reading. The water concentration in [EMIM][MeSO_3_]/water solutions was measured using the Karl Fisher Titrator (Mettler Toledo™ C20D) with a relative uncertainty less than 0.5%. A magnetic stirrer (Thermo Scientific Cimarec Micro Stirrers) was used to stir the [EMIM][MeSO_3_]/water solution inside the reactant bottle during the VLE measurement. The data logging and the conversion from RH and *T* to water vapor pressure were performed using the HW4-E software in a computer.

### VLE measurements

Twelve [EMIM][MeSO_3_]/water solutions with molar concentration of water from 18% to 92% were prepared in the VLE measurements. For each solution, its RH was measured at temperatures 303 K, 323 K, 343 K, and 363 K. The sample solution with an approximate volume of 100 ml was placed in the reactant bottle. The oven temperature was set to the desired temperature, and the sample solution was heated and stirred vigorously with magnetic stirrers to get homogeneous mixing. At the same time, the data logging was started. After the system reached the equilibrium and the RH stayed unchanged for 30 minutes, the equilibrium temperature and RH were recorded. A series of equilibrium temperature and water vapor pressure (or RH) data were obtained for the [EMIM][MeSO_3_]/water solutions.

## Results and Discussion

### Activity coefficient of water

**Figure 2:**
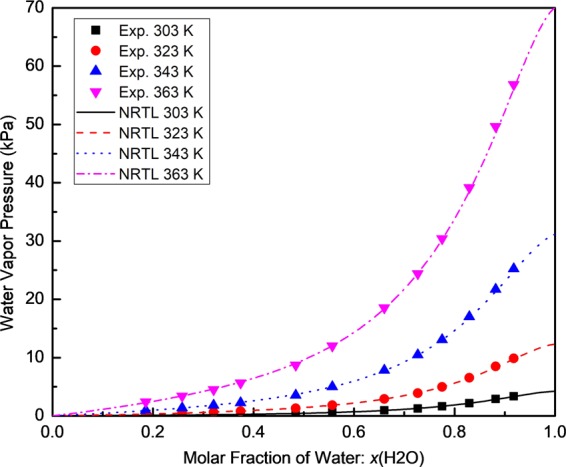
Water vapor pressure in the [EMIM][MeSO_3_]/water binary solutions versus the molar fraction of water in the temperature range of 303 K to 363 K.

The experimental VLE data for the [EMIM][MeSO_3_]/water binary solutions with water molar concentrations from 18% to 92% were measured and listed in Table 1. The vapor pressure ${p}_{{{\rm{H}}}_{2}{\rm{O}}}$ were determined using the measured RH. The relative uncertainty of the measured mole fraction *x*(H_2_O) was found to be less than 5.02% using Eq. (12). The RH and *T* were measured simultaneously using the humidity sensor with an uncertainty of *T* = ±0.1 K and RH = ±0.8% of the RH reading. Figure 2 shows the measured water vapor pressure in [EMIM][MeSO_3_]/water binary solutions versus the molar fraction of water in a temperature range of 303 K to 363 K.

**Table 1:** Experimental VLE data of the[EMIM][MeSO_3_]/water binary solutions at different temperatures.

*x*(H_2_O)	Relative Humidity (%)			
	303 K	323 K	343 K	363 K
0.1855	2.31	2.68	3.04	3.44
0.2579	3.46	3.89	4.36	4.86
0.3207	4.87	5.40	6.00	6.67
0.3744	5.98	6.57	7.28	8.05
0.4840	9.86	10.60	11.47	12.45
0.5568	13.99	14.90	16.02	17.14
0.6602	22.42	23.71	25.14	26.45
0.7269	30.19	31.74	33.60	34.80
0.7752	38.94	40.33	42.00	43.35
0.8294	51.55	53.12	54.63	55.89
0.8821	68.20	68.84	69.76	70.82
0.9178	79.35	80.12	81.00	81.06
***x*(H**_**2**_**O)**	**Water Vapor Pressure (Pa)**			
	**303** **K**	**323** **K**	**343** **K**	**363** **K**
0.1855	98.01	330.71	947.63	2412.03
0.2579	146.81	480.03	1359.10	3407.69
0.3207	206.63	666.36	1870.32	4676.80
0.3744	253.73	810.74	2269.32	5644.42
0.4840	418.36	1308.04	3575.43	8729.57
0.5568	593.60	1838.66	4993.75	12018.05
0.6602	951.28	2925.81	7836.64	18545.95
0.7269	1280.96	3916.72	10473.79	24400.72
0.7752	1652.22	4976.72	13092.24	30395.72
0.8294	2187.27	6555.01	17029.26	39188.39
0.8821	2893.73	8494.86	21745.59	49656.86
0.9178	3366.82	9886.81	25249.32	56836.84

**Figure 3:**
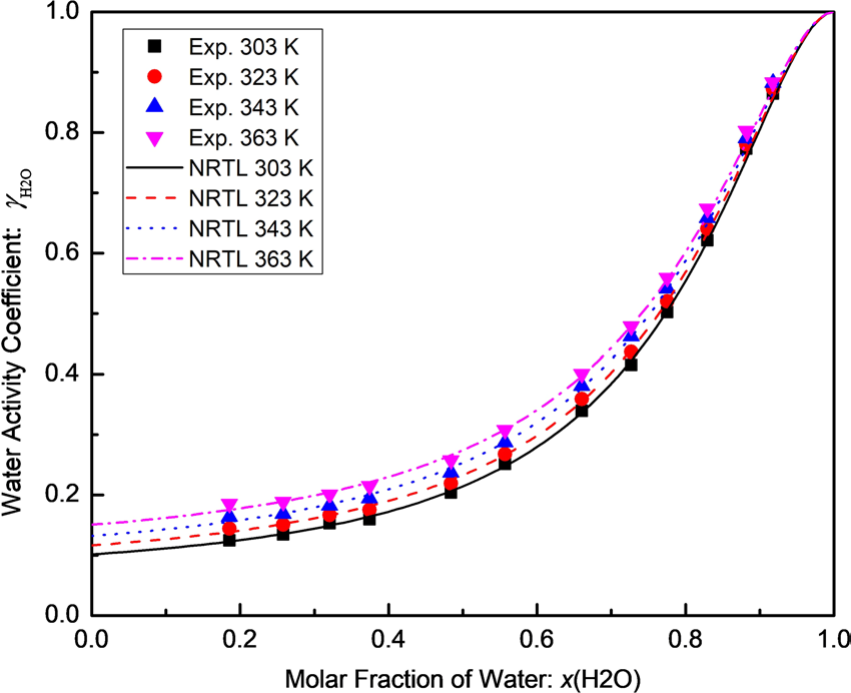
Activity coefficient of water in the [EMIM][MeSO_3_]/water binary solutions versus the molar fraction of water in the temperature range of 303 K to 363 K.

The effect of the ionic liquid on the non-ideality of the aqueous solutions can be expressed by the activity coefficients of water ${\gamma }_{{{\rm{H}}}_{2}{\rm{O}}}$, which was calculated by Eq. (1). Table 2 shows the calculated activity coefficients of water ${\gamma }_{{{\rm{H}}}_{2}{\rm{O}}}$ in the [EMIM][MeSO_3_]/water binary solutions. The relative uncertainty of the water activity coefficient ${\gamma }_{{{\rm{H}}}_{2}{\rm{O}}}$ was found to be 5.09%. The plot of the activity coefficient ${\gamma }_{{{\rm{H}}}_{2}{\rm{O}}}$ versus the molar concentration of water is shown in Fig. 3. As shown in this figure, the water activity coefficient ${\gamma }_{{{\rm{H}}}_{2}{\rm{O}}}$ approaches a value of one when the water concentration is close to 100%, as expected by Raoult’s law. For water concentrations below about 30 mol.%, ${\gamma }_{{{\rm{H}}}_{2}{\rm{O}}}$ approaches almost constant values between 0.10 and 0.15. A small value of ${\gamma }_{{{\rm{H}}}_{2}{\rm{O}}}\,$indicates a large deviation from the ideal solution behavior or from Raoult’s law. A small value of ${\gamma }_{{{\rm{H}}}_{2}{\rm{O}}}\,$is desired for the application in gas dehydration. The influence of the temperature on ${\gamma }_{{{\rm{H}}}_{2}{\rm{O}}}$ is small in the temperature range of test, especially for low water concentrations. For a given water concentration, ${\gamma }_{{{\rm{H}}}_{2}{\rm{O}}}$ increases slightly when the temperature increases, which is consistent with the temperature dependence of the interaction energy $\Delta {g}_{12-11}$ in the [EMIM][MeSO_3_]/water binary solutions.

**Table 2:** Water Activity Coefficient ${{\boldsymbol{\gamma }}}_{{{\rm{H}}}_{2}{\rm{O}}}$ of the [EMIM][MeSO_3_]/water binary solutions at different temperatures.

*x*(H_2_O)	Water Activity Coefficient ${\gamma }_{{{\rm{H}}}_{2}{\rm{o}}}$			
	303 K	323 K	343 K	363 K
0.1855	0.1245	0.1445	0.1639	0.1854
0.2579	0.1342	0.1509	0.1691	0.1885
0.3207	0.1518	0.1684	0.1871	0.2080
0.3744	0.1597	0.1755	0.1944	0.2150
0.4840	0.2037	0.2190	0.2370	0.2572
0.5568	0.2512	0.2676	0.2877	0.3078
0.6602	0.3396	0.3592	0.3808	0.4007
0.7269	0.4153	0.4366	0.4622	0.4787
0.7752	0.5023	0.5202	0.5418	0.5592
0.8294	0.6215	0.6404	0.6586	0.6738
0.8821	0.7732	0.7804	0.7908	0.8029
0.9178	0.8645	0.8729	0.8825	0.8832

The water activity coefficients at infinite dilution ${\gamma }_{{{\rm{H}}}_{2}{\rm{O}},\infty }$ for the [EMIM][MeSO_3_]/water binary solutions are listed in Table 3. It is found that the limiting water activity coefficients ${\gamma }_{{{\rm{H}}}_{2}{\rm{O}},\infty }$ is in the range of 0.102 to 0.151 at temperatures from 303 K to 363 K, more than 4 times lower than that for triethylene glycol, which indicates that the ionic liquid [EMIM][MeSO_3_] possesses much stronger ability of absorbing water vapor than the commonly used triethylene glycol desiccants.

**Table 3:** Water activity coefficient at infinite dilution ${{\boldsymbol{\gamma }}}_{{{\rm{H}}}_{2}{\rm{O}},\infty }$ for the [EMIM][MeSO_3_]/water binary system at different temperatures.

Temperature (K)	303	323	343	363
${{\rm{\gamma }}}_{{{\rm{H}}}_{2}O,\infty }$	0.1015	0.1165	0.1322	0.1507

There exist some discrepancies in ${\gamma }_{{{\rm{H}}}_{2}{\rm{O}},\infty }$ in literature. For example, Krannich *et al*.^7^ reported the ${{\rm{\gamma }}}_{{{\rm{H}}}_{2}{\rm{O}},\infty }$ around 0.20 that was determined using the boiling point method. Domanska *et al*.^16^ reported the ${{\rm{\gamma }}}_{{{\rm{H}}}_{2}{\rm{O}},\infty }$ in the range 0.071~0.088, which were determined with the gas-liquid chromatography method. In this study, the limiting water activity coefficients ${\gamma }_{{{\rm{H}}}_{2}{\rm{O}},\infty }$ was found to be in the range of 0.102 to 0.151 at temperatures from 303 K to 363 K. The differences in the reported ${\gamma }_{{{\rm{H}}}_{2}{\rm{O}},\infty }$ data might be due to the different measurement techniques.

### Interaction energy between molecules and non-randomness parameters

The exchange in interaction energy $\Delta {g}_{12-11}$, and $\Delta {g}_{12-22}$ and the non-randomness parameter ${\alpha }_{12}$ in the [EMIM][MeSO_3_]/water binary solutions can be extracted by fitting the experimental data ${\gamma }_{{{\rm{H}}}_{2}{\rm{O}}}\,$to the NRTL equations (Eqs. (3–4)). These parameters are listed in Table 4. In Table 4, the subscript “1” represents water, while the “2” represents the ionic liquid [EMIM][MeSO_3_]. $\Delta {g}_{12-11}$ is the interaction energy change as a result of breaking an H_2_O-H_2_O interaction and forming a [EMIM][MeSO_3_]-H_2_O interaction. As shown in Table 4, the interaction energy parameters $\Delta g$ follow the order [EMIM][MeSO_3_]-[EMIM][MeSO_3_] > [EMIM][MeSO_3_]-H_2_O ≫ H_2_O-H_2_O. The large negative value of $\Delta {g}_{12-11}$ indicates that the intermolecular attractive force between [EMIM][MeSO_3_] and H_2_O is much stronger than that between H_2_O and H_2_O. The absolute value of interaction energy $\Delta {g}_{12-11}$ and $\Delta {g}_{12-22}$ decreases with increasing temperature, which could be attributed to the increasing thermal motion of the molecules^18^.

**Table 4:** Interaction Energy and Non-Randomness parameters for the [EMIM][MeSO_3_]/water binary system at different temperatures.

Temperature (K)	303	323	343	363
*α*_12_	0.425	0.461	0.492	0.535
$\Delta {{\rm{g}}}_{12-11}$ (J/mol)	−7428	−7401	−7379	−7284
$\Delta {{\rm{g}}}_{12-11}$ (J/mol)	2559	2490	2450	2405
${g}_{11}$ (J/mol)	−41270	−40240	−39210	−38150
${g}_{12}$ (J/mol)	−48698	−47641	−46589	−45434
${g}_{22}$ (J/mol)	−51257	−50131	−49039	−47839
ARD	2.22%	2.64%	2.98%	3.13%

For comparison, the exchange in interaction energy $\Delta {g}_{12-11}$ and $\Delta {g}_{12-22}$ in some other ionic liquids/water binary solutions were summarized in Table 5 ^19,20,24,25^. It should be noted that the $\Delta {g}_{12-11}$ in the [EMIM][MeSO_3_]/water binary solution was found to be larger than that in 1-ethyl-3-methylimidazolium ethylsulfate ([EMIM][EtSO_4_])/water solution^25^, which is another promising ionic liquid for moisture removal and shares similar chemical structure^26,27^. The difference in interaction energy may result from the shorter alkyl group in [EMIM][MeSO_3_] anion, which is favorable for the bonding with water molecule^28,29^.

**Table 5:** Interaction Energy parameters for other ionic liquid/water binary systems.

Ionic Liquids	Δ*g*_12−11_ (J/mol)	Δ*g*_12−22_ (J/mol)
[EMIM][(CF_3_SO_2_)_2_N]^19^	−1458	12913
[BMIM][(CF_3_SO_2_)_2_N]^19^	−349	19436
[EMIM][DMP]^20^	−3925	5278
[MMIM][DMP]^24^	−9566	5065
[EMIM][DEP]^24^	−6535	11818
[EMIM][EtSO_4_]^25^	−1440	4439

The interaction energy between water molecules ${g}_{11}$ is the molar vaporization energy of water (i.e., cohesive energy) but has a negative sign on it^30^, which is available in liteature^31^. The interaction energy between the ionic liquid and water molecules ${g}_{12}$ can be calculated using the formula: ${g}_{12}=\Delta {g}_{12-11}+{g}_{11}$. Similarly, the interaction energy between the ionic liquid molecules ${g}_{22}$ can be determined by ${g}_{22}=\Delta {g}_{12-22}+{g}_{12}$. The obtained interaction energies ${g}_{11},\,{g}_{12}$ and ${g}_{22}$ are summarized in Table 4. These molecular interaction energies have negative signs due to the intermolecular attractive forces. It is found in Table 4 that the molecular interaction energies become less negative when the temperature increases. The magnitude of the interaction energy between the [EMIM][MeSO_3_] and water molecules was found to be in the range of 45~49 kJ/mol, which was 20% larger than that between the water molecules in the [EMIM][MeSO_3_]/water system. The large interaction energy between the ionic liquid [EMIM][MeSO_3_] and water molecules can explain many reported macroscopic thermodynamic properties, such as small water activity coefficient and strong hygroscopicity.

The parameters ${\alpha }_{12}$ is related to the non-randomness in the liquid mixture; when ${\alpha }_{12}$ is zero, the local distribution around the center molecule is completely random. The non-randomness parameters ${\alpha }_{12}$ in the [EMIM][MeSO_3_]/water binary solutions were found to be around 0.5, as shown in Table 4. The values of *α*_12_ are generally consistent with those reported for other water/hygroscopic ionic liquid binary solutions^17,20,24^. The non-zero *α*_12_ in the [EMIM][MeSO_3_]/water binary solutions are mainly due to the difference in interaction energy and size between water and [EMIM][MeSO_3_].

The extent of the correlation between the experimental data and the NRTL model was evaluated by calculating the absolute relative deviation (ARD)^24^:

13 $${\rm{ARD}}=\frac{\sum |\frac{{{\rm{\gamma }}}_{\exp }-{{\rm{\gamma }}}_{{\rm{NRTL}}}}{{{\rm{\gamma }}}_{\exp }}|}{{\rm{n}}}$$

where n is the number of data points, ${\gamma }_{\exp }$ is the *γ* value calculated from experimental data, and ${\gamma }_{{\rm{NRTL}}}$ is the *γ* value calculated from the NRTL model. The values of ARD are also listed in Table 4, which implies a satisfactory correlation in the test temperature range.

### Heat of desorption

One application of these molecular interaction energies is that they can be used to determine the heat of desorption of the aqueous ionic liquid solutions. The internal energy in the aqueous ionic liquid solution *U*_*l*_ is the sum of the excess internal energy of the solution *U*^*E*^ and the molar internal energy of the pure component *U*_*i*_:

14 $${U}_{l}={U}^{E}+{n}_{1}{U}_{1}+{n}_{2}{U}_{2}$$

where *n*_1_ and *n*_2_ are the mole number of component 1 (i.e., water) and component 2 (i.e., ionic liquid), respectively. In the evaporation process, the intermolecular interaction energy is dominant, and therefore the intramolecular interaction energy can be neglected in the internal energy. The excess internal energy of the aqueous ionic liquid solution *U*^*E*^ can be evaluated as^18,32^:

15 $${U}^{E}={(g}^{(1)}-{g}_{11})\,{n}_{1}+{(g}^{(2)}-{g}_{22})\,{n}_{2}$$

where ${g}_{11}$ and ${g}_{22}$ are the molar interaction energy between water molecules and between the ionic liquid and water molecules, respectively, and ${g}^{(1)}$ and ${g}^{(2)}$ represent the molar residual Gibbs energy for molecule cells having component 1 and component 2 at the center respectively^33^. ${g}^{(1)}$ and ${g}^{(2)}$ can be calculated as:

16 $${g}^{(1)}={x}_{11}{g}_{11}+{x}_{21}{g}_{12}={g}_{11}+{x}_{21}\Delta {g}_{12-11}$$

17 $${g}^{(2)}={x}_{12}{g}_{12}+{x}_{22}{g}_{22}={g}_{22}+{x}_{12}\Delta {g}_{12-22}$$

where *x*_11_, *x*_22_, *x*_21_ and *x*_12_ represent the local mole fractions. For example, *x*_21_ is the local mole fraction of component 2 around the center component 1. *x*_21_ and *x*_12_ depend on the global concentration according to the NRTL model,

18 $${x}_{21}=\frac{{x}_{2}\ast exp(\,-\,{\alpha }_{12}\Delta {g}_{12-11}/RT)}{{x}_{1}+{x}_{2}\ast exp(\,-\,{\alpha }_{12}\Delta {g}_{12-11}/RT)}=\frac{{n}_{2}\ast exp(\,-\,{\alpha }_{12}\Delta {g}_{12-11}/RT)}{{n}_{1}+{n}_{2}\ast exp(\,-\,{\alpha }_{12}\Delta {g}_{12-11}/RT)}$$

19 $${x}_{12}=\frac{{x}_{1}\ast \exp (\,-\,{\alpha }_{12}\Delta {g}_{12-22}/RT)}{{x}_{2}+{x}_{1}\ast \exp (\,-\,{\alpha }_{12}\Delta {g}_{12-22}/RT)}=\frac{{n}_{1}\ast \exp (\,-\,{\alpha }_{12}\Delta {g}_{12-22}/RT)}{{n}_{2}+{n}_{1}\ast \exp (\,-\,{\alpha }_{12}\Delta {g}_{12-22}/RT)}$$

Differentiating Eq. (14) leads to the change of internal energy in the liquid solution for the evaporation of one mole of water:

20 $$\Delta {U}_{l}=-{g}_{11}-\frac{\Delta {g}_{12-11}\ast {{x}_{2}}^{2}\ast exp(-2{\alpha }_{12}\Delta {g}_{12-11}/RT)}{{({x}_{1}+{x}_{2}\ast exp(-{\alpha }_{12}\Delta {g}_{12-11}/RT))}^{2}}-\frac{\Delta {g}_{12-22}\ast {{x}_{2}}^{2}\ast \exp (-{\alpha }_{12}\Delta {g}_{12-22}/RT)}{{({x}_{2}+{x}_{1}\ast \exp (-{\alpha }_{12}\Delta {g}_{12-22}/RT))}^{2}}$$

Considering the volume work in the evaporation process, which is equal to *RT* based on the ideal gas assumption, the heat of desorption can be calculated using the interaction energies:

21 $$\begin{array}{ccc}\Delta {H}_{v} & = & -{g}_{11}+RT-\frac{\Delta {g}_{12-11}\ast {{x}_{2}}^{2}\ast exp(-\,2{\alpha }_{12}\Delta {g}_{12-11}/RT)}{{({x}_{1}+{x}_{2}\ast exp(-{\alpha }_{12}\Delta {g}_{12-11}/RT))}^{2}}\\  &  & -\,\frac{\Delta {g}_{12-22}\ast {{x}_{2}}^{2}\ast \exp (-\,{\alpha }_{12}\Delta {g}_{12-22}/RT)}{{({x}_{2}+{x}_{1}\ast \exp (-{\alpha }_{12}\Delta {g}_{12-22}/RT))}^{2}}\end{array}$$

The formula can be used to predict the heat of desorption in the aqueous binary solutions when the interaction energies are given.

**Figure 4:**
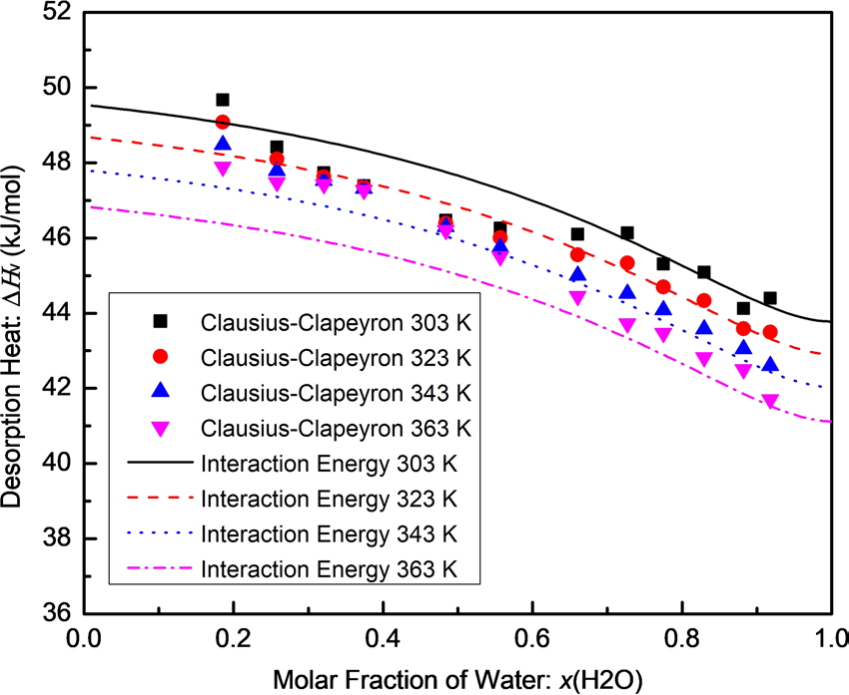
Desorption heat calculated from the interaction energy in the temperature range of 303 K to 363 K and water fraction range of 0% to 100%. (The desorption heat calculated by the Clausius-Clapeyron Equation is also shown for comparison).

Figure 4 shows the heat of desorption calculated from the interaction energies in the [EMIM][MeSO_3_]/water binary solutions with water fraction from 0% to 100% at temperatures 303 K, 323 K, 343 K, and 363 K. The desorption heat calculated by the Clausius-Clapeyron Equation is also shown for comparison. As shown in Fig. 4, they are in good agreement. The desorption heat $\Delta {H}_{v}$ decreases with increasing temperature for a given water concentration. This trend is consistent with the temperature dependence of the interaction energy parameters $\Delta {g}_{12-11}$ and $\Delta {g}_{12-22}$ listed in Table 4, which could be attributed to the increasing thermal motion of the molecules at elevated temperatures. Due to the strong bonding forces between water and [EMIM][MeSO_3_], the desorption heat or enthalpy of vaporization of water in the [EMIM][MeSO_3_]/water solutions is always higher than the enthalpy of vaporization of pure water at the same temperature, but the difference becomes smaller when the water concentration approaches 100%.

## Conclusion

In this work, molecular thermodynamic properties such as interaction energies and non-randomness parameter of the [EMIM][MeSO_3_]/water binary system were determined from the water activity coefficient data using the Non-Random Two-Liquid (NRTL) model. The water activity coefficient of this binary system was measured with molar concentrations of water from 18% to 92% at temperatures 303 K, 323 K, 343 K, and 363 K. The interaction energy between the ionic liquid [EMIM][MeSO_3_] and water molecules (${g}_{12}$) was found to be ~20% larger than that between the water molecules (${g}_{11}$). The exchange in interaction energy $\Delta g$ followed the order [EMIM][MeSO_3_]-[EMIM][MeSO_3_] > [EMIM][MeSO_3_]-H_2_O» H_2_O-H_2_O. The large negative value of $\Delta {g}_{12-11}$ (−7427.51 J/mol to −7283.79 J/mol) indicated that the intermolecular attractive force between [EMIM][MeSO_3_] and H_2_O was much stronger than that between H_2_O and H_2_O. This can explain the observed strong hygroscopicity in the ionic liquid [EMIM][MeSO_3_]. With the molecular interaction energies, the heat of desorption was predicted in the [EMIM][MeSO_3_]/water binary system. The obtained heat of desorption was in good agreement with that calculated from the conventional Clausius-Clapeyron Equation.

## Data Availability

All data generated or analyzed during this study are included in this published article.
